# Feasibility of Non-Invasive Vagus Nerve Stimulation (gammaCore VET™) for the Treatment of Refractory Seizure Activity in Dogs

**DOI:** 10.3389/fvets.2020.569739

**Published:** 2020-09-16

**Authors:** Kelsey Robinson, Simon Platt, Georgina Stewart, Lisa Reno, Renee Barber, Lindsay Boozer

**Affiliations:** ^1^Department of Small Animal Medicine and Surgery, College of Veterinary Medicine, University of Georgia, Athens, GA, United States; ^2^Summit Veterinary Referral Center, Tacoma, WA, United States; ^3^Friendship Hospital for Animals, Washington, DC, United States

**Keywords:** vagus nerve stimulation, VNS, non-invasive, canine epilepsy, seizures, refractory epilepsy

## Abstract

Idiopathic epilepsy is the most common chronic neurologic condition in dogs. Approximately 20–30% of those dogs are refractory to standard medical therapy and commonly experience side effects from antiepileptic drugs. Non-invasive vagus nerve stimulation (nVNS) has been frequently used in human medicine as an adjunct seizure therapy with low incidence of adverse events. Canine studies are limited to invasive surgical implants with no non-invasive evaluations currently published. We investigated the feasibility and efficacy of nVNS (gammaCore VET) as an adjunct treatment for refractory epilepsy in dogs. In total, 14 client-owned dogs completed the trial of either 8- or 16-week treatment periods during which they received 90–120 s stimulation three times per day in the region of the left cervical vagus nerve. Owners recorded seizure type (focal or generalized) and frequency as well as any adverse effects. Out of 14 dogs, nine achieved a reduction in seizure frequency and four were considered responders with a 50% or greater reduction in seizures from baseline to the final treatment period. However, there was no statistically significant difference in overall seizure frequency (*p* = 0.53) or percent change in seizure frequency between groups (*p* = 0.75). Adverse effects occurred in 25% of dogs originally enrolled, with reports of a hoarse bark and limb trembling, lethargy, behavioral changes, and an increase in seizure frequency. Non-invasive VNS was found to be safe and easy to administer with mild adverse events. It is considered a feasible treatment option as an adjunct therapy in refractory seizures and should be further investigated.

## Introduction

Idiopathic epilepsy is the most common chronic neurological condition in dogs, with reported prevalence ranging from 0.5 to 5% (1–3). Of dogs with idiopathic epilepsy, 20–30% are refractory to treatment with standard antiepileptic medications, and less than half are able to maintain a seizure-free status without experiencing side effects (4, 5). These patients may have multiple seizure episodes over short time periods, contributing to primary brain injury from excitotoxic and metabolic derangements as well as secondary systemic sequelae such as respiratory and cardiac effects (6). Alternative therapies are needed for these dogs in order to achieve better seizure control, minimize adverse effects, and consequently improve quality of life for both the dog and owner.

Vagus nerve stimulation (VNS) may serve as an alternative or adjunct therapy for dogs with refractory epilepsy. This method has been utilized in human medicine for treatment of various diseases, including pharmacoresistent epilepsy and status epilepticus (7, 8). Side effects attributed to the implantable device in humans are minimal, most often causing a voice change and coughing, (7) although there have been more severe documented complications related to the surgical procedure used to implant the device. Clinical evaluation of a surgically implanted vagus nerve stimulator in dogs found up to 50% reduction in seizure frequency in four of nine dogs (9).

Despite potentially promising results, vagus nerve stimulator implantation remains uncommon in canine patients, likely due to the expense and the expertise required for placement. More recently, non-invasive vagus nerve stimulation (nVNS) has been reported in human literature, which has been found to be safe and tolerable in its use in adjunct therapy for epilepsy, migraines and cluster headaches, multiple psychiatric disorders, and pain syndromes (10–17).

The purpose of this pilot study was to evaluate the feasibility of a hand-held non-invasive vagus nerve stimulator (gammaCore VET) as a treatment for refractory epilepsy in dogs. It was hypothesized that the treatment would be safe and easy to administer, and would result in a decrease in seizure frequency and severity when used three times daily. We aimed to evaluate efficacy in seizure reduction and any adverse effects that may occur, as well as the feasibility of owner administration.

## Materials and Methods

### Study Population

Dogs with refractory idiopathic epilepsy were recruited from 2014 to 2018. Dogs must have met either tier I or tier II confidence levels for idiopathic epilepsy as designated by the International Veterinary Epilepsy Task Force (IVETF). For tier I, this includes an onset of unprovoked epileptic seizures between 6 months and 6 years of age, with two or more seizures occurring at least 24 h apart. They must also have had a normal physical and neurological examination in the inter-ictal period and an unremarkable workup including complete blood count, serum chemistry, and urinalysis. Tier II confidence of diagnosis includes the previous factors, as well as unremarkable pre- and post-prandial bile acids, magnetic resonance imaging (MRI) of the brain, and cerebrospinal (CSF) analysis. Additionally, patients must have had a minimum 6-month history of generalized seizures with or without focal seizures with no fewer than two seizures per month for the most recent 2 months with no abnormalities on neurological exam.

Patients were considered refractory if they had been maintained on phenobarbital (PB) for 2 months and/or potassium bromide (KBr) for 6 months with no successful improvement in seizure activity (defined as <50% decrease in seizures compared to initial frequency). Dogs were excluded if there was evidence of systemic illness, particularly cardiac disease or severe skin disease affecting the area where the nVNS device would be placed for stimulation. Dogs requiring emergency treatment for status epilepticus or cluster seizures remained within the study, but dogs with subsequent changes in long-term therapy were thereafter removed from the trial.

The study was approved by the University of Georgia's College of Veterinary Medicine clinical research committee (approval number CR-367). Owner consent and information forms were provided and signed by the owners.

### nVNS Device

**Figure 1:**
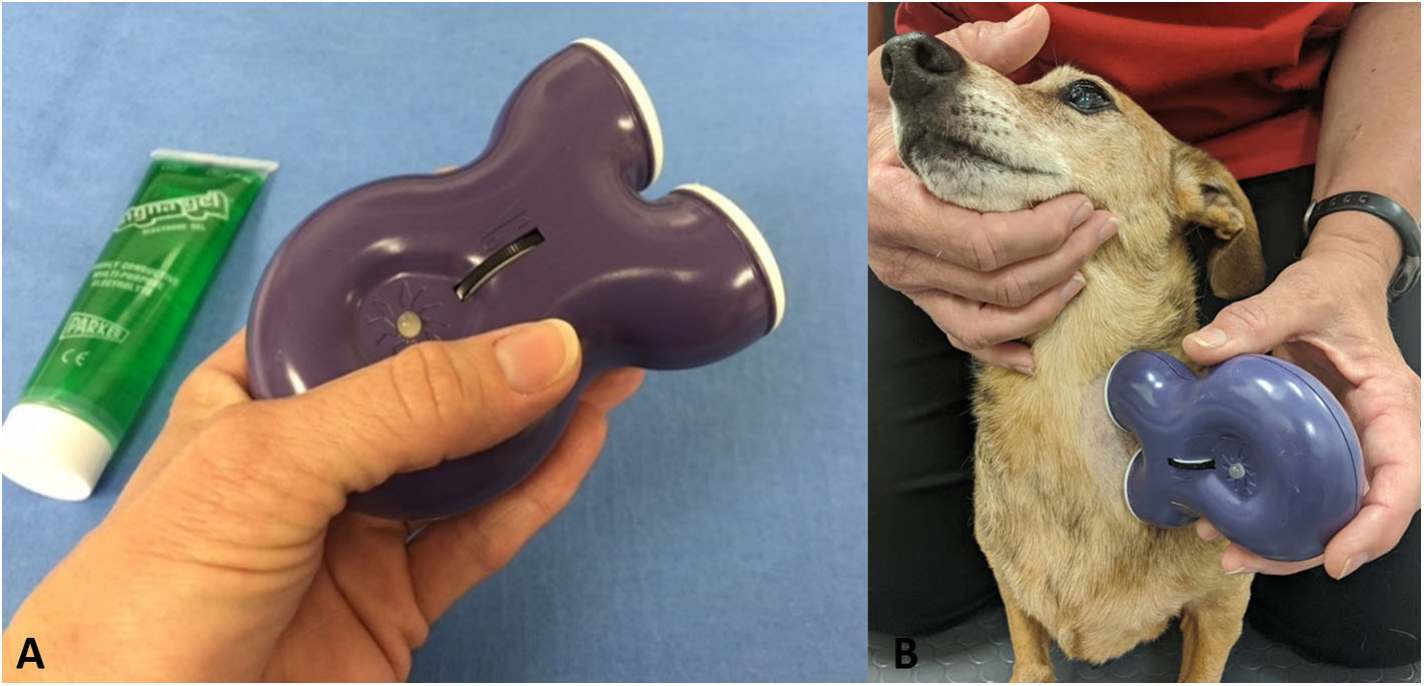
**(A)** GammaCore VET device. **(B)** Application of device to left cervical area with a small amount of conductive gel.

The gammaCore VET device produces a signal consisting of five 5,000 hertz (Hz) pulses repeated at a rate of 25 Hz for a maximum of 120 s per stimulation. The waveform of the electric pulses approximates a sine wave with peak voltage limited to 24 volts (V) when against the skin of the neck and a maximum output current of 60 milliamperes (mA). The amplitude of the stimulation is adjusted by using the thumbwheel located on the device, which ranges from 1 to 5. The device is shown in Figure 1A.

### Treatment Protocol

Owners were provided with a handheld gammaCore VET device and instructed on its use. The hair was clipped on the left side of the neck of each dog, and owners were instructed to provide vagus nerve stimulation three times daily by applying conductive gel to the shaved area, holding up the device, and stimulating for 90–120 s (Figure 1B). It was recommended to place the device at an intensity of 2.5/5, but this was allowed to be changed based on the dog's tolerance.

Each dog underwent an 8-week baseline assessment during which seizure type and frequency were recorded by the owner on standard forms. Following this baseline assessment, dogs were randomly assigned to two groups. Group 1 received nVNS treatments for the first 8-week period followed by a second 8-week period of continued treatment. Group 2 received no change in their current treatment for the first 8-week period, followed by an 8-week period of nVNS treatment.

Dosage of pre-existing anticonvulsant treatment was not altered throughout the study. Dogs were examined at 8, 16, and 24 weeks after randomization. At each of these visits, the record of seizure activity and adverse effects were reviewed by an investigator blinded to patient treatment group. Complete blood count, serum chemistry profile, urinalysis, bile acids, and serum anticonvulsant (PB or KBr) concentrations were performed at the initial screening visit and at week 24. No monitoring of zonisamide (ZNS) or levetiracetam (LEV) serum levels was performed due to lack of established effective ranges in dogs.

Seizure frequency, adverse effects, and serum anticonvulsant levels relative to baseline were compared between the treatment period and the control period. Any patients having unacceptable adverse effects or needing change to long term therapy during the study were withdrawn and treated appropriately.

### Outcome Measures

The goals of this study were to evaluate the efficacy, adverse effects, and feasibility of adjunct nVNS in dogs with refractory idiopathic epilepsy. The primary outcome, change in seizure frequency, was measured by comparing the frequency changes during each time period within the trial. The seizure frequency for each time period in the trial was calculated by dividing the total number of seizures in that period by 8 weeks. Dogs were considered responders if there was a reduction in seizure frequency by at least 50% during weeks 17–24 compared to baseline frequency during weeks 1–8. Change in the frequency of types of seizures (focal or generalized) and adverse events were considered as secondary outcome measures. Lastly, we were interested in owner compliance with treatment and feasibility of administration.

### Statistical Analysis

Due to small sample size, non-parametric statistical tests were utilized. The Mann Whitney *U*-test was used for between group comparisons of age and weight, a Fisher's exact test was used for comparison of method of diagnosis and reproductive status, and a Wilcoxon signed rank test was used to compare pre- and post-therapy antiepileptic drug levels. The primary outcome was evaluated by a Mann Whitney *U*-test for comparison of the median change in mean seizure frequency during weeks 1–8 and weeks 17–24 for all dogs, as well as by a Friedman test for change in total seizure frequency across all time points in each group. A Friedman test was also applied to the change in frequency of seizure types across all time points in each group. The Mann Whitney *U*-test was also used for comparison of percent change in seizure frequency for each group to identify any differences in response rate with treatment length. Statistical software (JMP, SAS Institute, Cary, NC) was utilized for these procedures.

## Results

### Animals

A total of 16 dogs were recruited for enrollment. There were nine dogs assigned to group 1 and seven dogs assigned to group 2. Two patients were withdrawn from the study—one due to behavioral side effects and the second due to an increase in seizure frequency which the owners felt was unacceptable. Because there was no data for additional analysis, these dogs were not considered in the outcome measures except in initial patient demographics and reporting adverse events. The total number of patients completing the trial was 14, with eight in group 1 and six in group 2. There was no significant difference between groups, including breed, age, sex, or confidence level of diagnosis, which is shown in Table 1. There was no significant difference in pre- and post-therapy serum PB levels (*p* = 0.12). No evaluation of KBr levels was performed due to low sample sizes.

**Table 1:** Patient demographics for all dogs recruited in group 1 vs. group 2.

**Characteristic**	**Group 1 (*n* = 9)**	**Group 2 (*n* = 7)**	***p*-value**
Median age at start of study (range), years	6 (range 3–10 years)	4 (range 2–8 years)	0.63
Median weight (range), kg	36.3 (6.2–47.2)	32 (4.6–39)	0.30
Sex	MN (7), MI (1), FS (1)	MN (4), FS (3)	0.26
Diagnosis	Tier I (4) Tier II (5)	Tier I (4) Tier II (3)	>0.99
Breed	Golden retriever (3), Mixed breed (2), Boston terrier (1), Chihuahua (1), Labrador retriever (1), Doberman pincher (1)	Mixed breed (3), Golden retriever (1), Yorkshire terrier (1), Collie (1), Greyhound (1)	-
AED Therapy	1–PB, KBr, ZNS, LEV 2–PB, KBr, ZNS 3–PB, KBr, LEV 4–KBr, ZNS 5–PB, KBr, LEV 6–PB, LEV 7–PB, KBr, ZNS, LEV 8–KBr, ZNS, LEV 9–PB, KBr, ZNS, LEV, hemp oil	1–PB, KBr 2–PB, KBr, ZNS 3–PB, KBr, LEV 4–PB, ZNS, LEV 5–KBr, LEV 6–KBr, LEV 7–PB	-

### Change in Seizure Frequency

Out of 14 dogs, four were considered responders. In total, 9/14 dogs had a reduction in seizure frequency, 1/14 had no change, and 4/14 experienced an increase in seizure frequency. There was no significant difference in the seizure frequency at baseline (weeks 1–8) to that of the final trial period (weeks 17–24) at *p* = 0.53. A summary of the number of dogs per group in each category, as well as a comparison of the median seizure frequency of each group compared to each other is shown in Table 2.

**Table 2:** Comparison of median change in seizure frequency/week and the median percent change in with corresponding *p*-values.

	**Group 1**	**Group 2**	***p*-value**
Median change in freq/wk (range)	−0.1875 (−14.25 to 1.625)	−0.4375 (−1.0 to 0.875)	0.9
Median percent change (range)	−25.89% (−100 to 130%)	−31.37% (−100 to 175%)	0.75
Responders	3	1	-
Decrease in freq	5	4	-
Increase in freq	2	2	-
No change	1	0	-

*Also shown are the number of dogs per group that were responders, and those with an increase, decrease, or no change in seizure frequency. There were no significant differences*.

**Figure 2:**
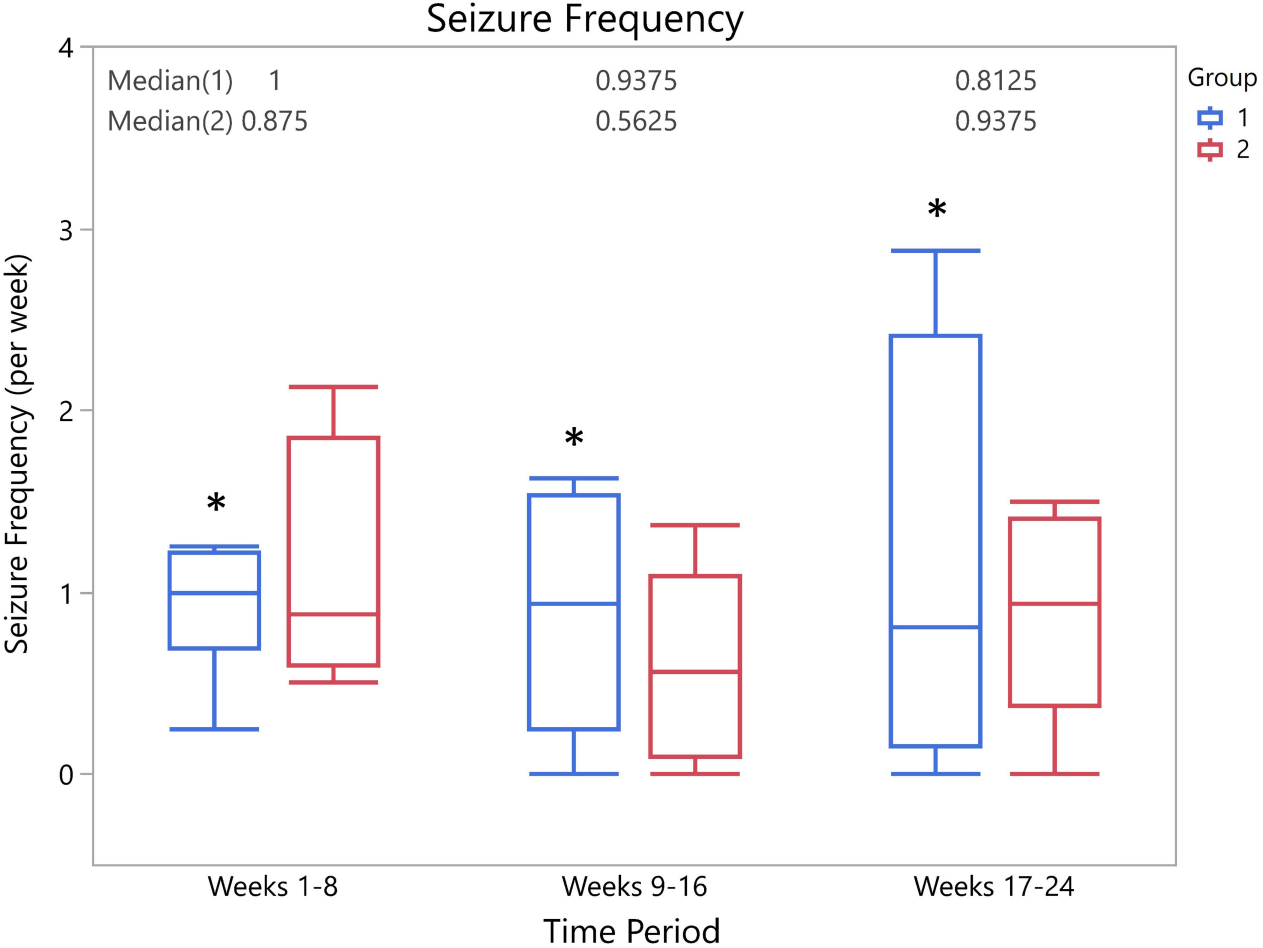
Box-and-whisker plots of median seizure frequency for each group at each time period. There was no significant difference in seizure frequencies between groups at any time point (weeks 1–8, *p* = 0.9; weeks 9–16, *p* = 0.4; weeks 17–24, *p* = 1.0). *Indicates presence of a single outlier.

There was no significant difference between the seizure frequency across time points for group 1 (Friedman's *Q* = 1.3125, *p* = 0.52) or for group 2 (Friedman's *Q* = 3.5833, *p* = 0.17). A total of 12/14 owners provided full information about seizure type. There was no significant difference in the change in frequency of type of seizure in either group 1 (generalized Friedman's *Q* = 2.2143, *p* = 0.33; focal Friedman's *Q* = 0.0714, *p* = 0.96) or group 2 (generalized Friedman's *Q* = 1.2, *p* = 0.55; focal Friedman's *Q* = 1.3, *p* = 0.52). Median seizure frequencies at each time period for each group are shown in Figure 2.

There was an incomplete data point for a single phase (weeks 1–8) of the trial for one dog in group 2. This value was imputed conservatively at the lowest number of seizures required to qualify for the study (four total or two per month average). All seizure frequency data is available in the Supplementary Material.

### Adverse Events and Feasibility

Mild adverse events were reported in a total of four of the originally recruited 16 patients (25%). One dog experienced progressive behavioral changes that started almost immediately with nVNS treatment. The dog became withdrawn and nervous, which was considered very unusual and unacceptable by the owner, so he was removed from the study after 3 weeks of therapy. The other dog that was withdrawn from the study experienced an increase in seizure frequency involving once daily seizures with the start of nVNS, which was discontinued by the owner within 10 days. The remaining two patients experiencing adverse effects completed the trial. The first patient experienced a hoarse, less frequent bark as well as trembling of the left thoracic limb (on the side of treatment) which continually improved and was negligible by week 5 of treatment. The second dog was reported to be mildly lethargic following the initial week of treatment, which was not noted on any further treatments, and no other cause for this report was investigated. No other adverse effects were reported.

The stimulation intensity was reported in only 10/14 dogs, with the range spanning from 1 to 5, and some dogs receiving a variety of stimulation levels. Explanations from dog owners on chosen intensity were mostly absent, but were occasionally reported as changed based on tolerance; however, there was little information on how this decision was made and what behaviors a dog exhibited to be deemed intolerant.

The pet owners in this trial did not report any concerns when administering treatments and were able to give stimulations three times daily as requested. Regarding feasibility of administration, nVNS was reported to be simple and easy, and considered safe overall.

## Discussion

Non-invasive vagus nerve stimulation has been utilized in humans for adjunct seizure therapy with reports of seizure reduction rates ranging from 23 to 64.4% (11, 18–20). Our study evaluated nVNS with a non-invasive, handheld device (gammaCore VET) designed to deliver therapeutic signal to the left cervical vagus nerve for seizure reduction in refractory idiopathic epilepsy in dogs. The results found no significant difference in overall seizure frequency or frequency of types of seizure between time periods with and without nVNS treatment, though 9/14 dogs had a reduction in seizure frequency and of those, two dogs had no seizures following implementation of therapy. Adverse effects were relatively common (25%), but were mostly mild in nature with none being considered serious; however, one dog was withdrawn due to an increase in seizure frequency and did not complete the trial.

Because nVNS has not previously been evaluated in dogs, we aimed to establish feasibility as an adjunct therapy to traditional oral medication. Reported treatment regimens in humans vary widely for refractory epilepsy, ranging from 1 to 3 times daily for 15–240 min each (19–21). For other disease entities, including depression, cluster headaches, migraines, and asthma, the treatment regimen is even more diverse with some including single 90–120 s or 15 min treatments (10, 15, 22), pulses of 30–120 s 3–15 min apart (13, 23–25), and 2–3 times daily for 90–120 s (16, 26–28). Regarding other parameters used for nVNS, there are similarly varied protocols reported in a review of nVNS (29). The stimulation frequency ranges from 0.5 to 120 Hz, but is most commonly 20 or 25 Hz, and pulse widths range from 0.01 to 1 millisecond (ms), but were most commonly at 1 or 0.25 ms. The stimulation intensity, often determined by the patients themselves based on discomfort threshold, may be reported as amplitude (mA) and/or voltage (V). Reports go up to 60 mA or 24 V, but the final intensity setting is often not reported (29, 30).

The recommended use for the gammaCore device in humans is one cycle (120 s) three times daily for prevention of cluster headaches, with an increase to two cycles three times daily if needed (31). The veterinary specific device cycle is similar in that a cycle is 120 s long and there are adjustable settings from 1 to 5 with maximum output voltage at 24 V and current of 60 mA. We elected to carry out this study in a similar dosing manner; however, we aimed for at least 90-s treatment cycles, allowing for up to a 30 s grace period that could be required for positioning at the start of a cycle due to administration to a dog rather than self-administration. Dosing stimulation intensity was started arbitrarily at 2.5/5, which could be adjusted in either direction based on patient tolerance with owner reports of a range from 1 to 5 during the trial.

Within the vagus nerve are myelinated afferent axons which project to the nucleus of the solitary tract (NTS), followed by further projections to the brainstem and forebrain which modulate signaling to cortical and subcortical structures (32–36). This modulation is suggested to alter cerebral blood flow (CBF) as well as cause an increase of γ-aminobutyric acid (GABA) and noradrenaline (NE) levels, resulting in antiepileptic effects; however, these downstream modulations are not proven and are still debated (37–39). Functional magnetic resonance imaging (fMRI) for auricular nVNS has found changes consistent with an expected anticonvulsant effect, including decreased activation of limbic areas such as the amygdala and hippocampus, and increased activation of the thalamus, septum, and locus coeruleus (32, 36). Positron emission tomography (PET) in epileptic humans undergoing VNS has found altered CBF in both acute and chronic stimulation (within 20 h and 3 months later, respectively). During the immediate phase, there was decreased CBF in the bilateral hippocampi, amygdalae, and cingulate gyri as well as increased bilateral insular CBF, but these changes were not present at the chronic evaluation. For both time points, CBF increases were present in the bilateral thalami, hypothalami, inferior cerebellar hemispheres, and right post-central gyrus, indicating these areas may play a role in long term VNS seizure therapy (40).

A key difference in most human trials compared to ours is the stimulation location. In human trials on refractory epilepsy, the auricular branch of the vagus nerve is typically utilized for nVNS, as opposed to the left cervical stimulation that was performed in our veterinary patients. Although studies on epilepsy are primarily carried out using the auricular branch for nVNS, handheld devices including the gammaCore were developed specifically for use on the neck for cluster headaches and migraines (29). Upon comparison to invasive VNS (iVNS) placed on the left cervical vagus nerve, both auricular nVNS (32, 36) and cervical nVNS have been shown to have similar effects on the brain via fMRI in healthy human volunteers (32, 36, 41). With the knowledge of similar fMRI findings and variable success with surgically implanted stimulators in the cervical region, we believe application of nVNS to the left cervical region to be appropriate and more likely to be tolerated than stimulation of the ear in canine patients.

Adverse effects were reported in four of 16 dogs, and were considered mild in nature. Similar to reports in humans, one dog experienced a hoarse bark after treatment initiation (42). This dog also developed some trembling in the left thoracic limb, which improved with time and subsequent treatments. Another dog was reported to become withdrawn, which could be a fear behavior from the sensation of stimulation, or potentially some other behavioral modification as a result of the nVNS, though determining a definitive cause is challenging. A third patient withdrew after seizure frequency increased following treatment initiation. Lastly, one patient was noted to be possibly lethargic during the first week of treatment, though no investigation as to other causes was pursued, and the dog did not exhibit any other clinical signs throughout the remainder of the trial. Previous reports of adverse effects associated with iVNS in dogs included Horner syndrome ipsilateral to the side of implantation, seroma formation, and migration of the generator—all of which were transient and presumed to be related to the surgical implantation (9). Human patients undergoing nVNS have most commonly reported skin irritation (18.2%), headache (3.6%), and nasopharyngitis (1.7%), with only three identified serious adverse events in a total of 1,322 cases (29). Some common side effects which have been reported in association with the gammaCore specifically include: discomfort and redness or irritation of the application site, pain in the area of the face, head, and neck, including tooth ache, muscle twitching, and contractions of the face, head, or neck, facial droop or lip pull, headaches and migraines, dizziness, and tingle or prickling sensation of the skin when the device is applied. These side effects commonly resolve immediately upon discontinuation of stimulation (43).

Regarding client compliance and feasibility, there were no reports of difficulty in administering treatments by owners. Concerns with this treatment were based on the reported adverse events, and not on the ability to carry out the nVNS, though three times daily treatment may be difficult for some owners. In considering this overall, nVNS with the gammaCore VET appears to be a feasible treatment option.

This study has several limitations. Firstly, this was an open label study with no sham group for comparison. Although this could result in owner bias toward reporting of seizure frequency, we utilized the same seizure logs for all 24 weeks in which owners were to document any focal or generalized seizure activity throughout the study. Though we originally also intended to evaluate subjective parameters including severity of seizures, which may have been more likely to appear biased in the face of known treatment, too few owners reported these criteria and we were unable to provide these subjective data. Because we were interested in the feasibility of treatment, we feel that this early data provides a foundation upon which to build a larger study that can include a sham device to evaluate the efficacy of nVNS more thoroughly. Though we found no significant difference in seizure frequencies in our cases, these data are still generated from small samples and interpretation of true efficacy is difficult with these numbers. Additionally, many of our patients were refractory to standard treatment with known cluster seizure events and thus needed emergency seizure treatment. While patients were not disqualified if they received temporary emergency treatments, we required that no changes be made to long-term therapy, which likely discouraged some potential enrollments.

Another limitation is the arbitrary dose and time of administration for nVNS. Human studies vary greatly on the treatment regimen, so future investigations on the treatment efficacy with different dosage schedules or stimulation intensity would be warranted, though intensity variation is common in nVNS as it tends to be established by the patient's tolerance threshold. Lastly, we carried out our trial including nVNS over either 8- or 16-week time periods. In many human trials, efficacy has been shown to improve with longer treatment periods. One study found mean seizure reduction from baseline was 31.3 and 64.4% at 3 and 6 months, respectively (18). Another found significant reduction in median monthly seizure frequency at 6 and 12 months compared to baseline, and a significant difference from the control group only at the 12 month timepoint (21). A third found percent seizure reduction increased from 8, 16, and 24 weeks at 24, 34, and 38%, respectively (44), and a canine study found no difference in seizure frequency at baseline vs. treatment when considering the 13-week time periods as whole, but evaluation of the last 4 weeks found a significant reduction at 34.4% (9). A longer time period for treatment may be beneficial for higher reduction in seizure frequency, as we did not see a significant difference between our dogs which received 8 or 16 weeks of treatment.

In conclusion, nVNS appears to be a feasible adjunct therapy for refractory epilepsy in dogs, especially those already on multiple oral antiepileptic drugs or with intolerable adverse effects from those medications. This therapy was easy to administer and well-tolerated with a moderate frequency of adverse effects, which were mild in nature. Future studies with larger sample sizes and variations in treatment protocol are warranted.

## Data Availability Statement

All datasets generated for this study are included in the article/Supplementary Material.

## Ethics Statement

The animal study was reviewed and approved by The University of Georgia Clinical Research Committee. Written informed consent was obtained from the owners for the participation of their animals in this study.

## Author Contributions

SP contributed conception and design of the study. GS, LB, and RB assisted with enrolled case management. LR, GS, and LB obtained and organized data. KR performed statistical analysis and wrote first draft of the manuscript. All authors contributed to manuscript revision, read, and approved the submitted version.

## Conflict of Interest

The authors declare that the research was conducted in the absence of any commercial or financial relationships that could be construed as a potential conflict of interest. The reviewer DH declared a past co-authorship with one of the authors SP to the handling editor.
